# A case report of severe pirimiphos-methyl intoxication: Clinical findings and cholinesterase status

**DOI:** 10.3389/fphar.2022.1102160

**Published:** 2022-12-23

**Authors:** Tobias Zellner, Christian Rabe, Jens von der Wellen-Pawlowski, Dagmar Hansen, Harald John, Franz Worek, Florian Eyer

**Affiliations:** ^1^ Division of Clinical Toxicology and Poison Control Centre Munich, Department of Internal Medicine II, TUM School of Medicine, Technical University of Munich, Munich, Germany; ^2^ Bundeswehr Institute of Pharmacology and Toxicology, Munich, Germany

**Keywords:** organophosphate, pesticide, obidoxime, atropine, cholinesterase

## Abstract

A 63-year-old male was admitted to a district hospital after ingesting ethanol and pirimiphos-methyl (PM) with suicidal intentions. History included alcoholic cirrhosis with alcoholism, adiposity, diabetes with cerebral microangiopathy, chronic renal insufficiency, heparin-induced thrombocytopenia, and status post necrotizing fasciitis. Emergency medical service reported an alert patient without signs of cholinergic crisis; activated charcoal and atropine were administered. Upon hospital arrival, he received fluid resuscitation, activated charcoal, and atropine. He was transferred to a toxicology unit the next day. On admission, he had no cholinergic signs (dry mucous membranes, warm skin, and mydriatic pupils) requiring small atropine doses (0.5 mg per hour). Four hours after admission, he developed bradycardia and respiratory distress, necessitating intubation. He received atropine by continuous infusion for 7 days (248 mg total) and obidoxime (bolus and continuous infusion). PM, pirimiphos-methyl-oxon (PMO), and phosphorylated tyrosine (Tyr) adducts derived from human serum albumin were analyzed *in vivo*. Cholinesterase status (acetylcholinesterase (AChE), butyrylcholinesterase (BChE), inhibitory activity of patient plasma and reactivatability, and phosphorylated BChE-derived nonapeptides) was measured *in vivo*. Obidoxime and atropine were monitored. PM and PMO were detectable, PM with maximum concentration ∼24 h post admission (p.a.) and PMO at ∼18 h p.a. Tyr adducts were detectable. AChE *in vivo* was suppressed on admission, increased continuously after starting obidoxime, and reached maximum activity after ∼30 h. AChE *in vivo* and reactivatability remained at the same level until the end of monitoring. BChE was already suppressed on admission; termination of the antidote treatment was possible after BChE had recovered to 1/5th of its normal value and extubation was possible after BChE had recovered to 2/5th. While a substantial part of BChE was already aged on admission, aging continued peaking at ∼24 h p.a. After initiating obidoxime treatment, plasma levels increased until obidoxime plasma levels reached a steady state. On admission, plasma atropine level was low; it increased with the start of the continuous infusion. Afterward, the level dropped to a steady state. The clinical course was characterized by bouts of pneumonia, necessitating re-intubation and prolonged ventilation, sepsis, delirium, and a peripheral neuropathy. After psychiatric evaluation, the patient was discharged to a neurological rehabilitation facility after 77 days of hospital care.

## Introduction

Pirimiphos-methyl (PM) is an organophosphate (OP) which is used as an insecticide and marketed as Actellic® 50 by Syngenta (Syngenta. https, 2021). At the beginning of the millennium, OPs and other pesticides were responsible for 250,000–370,000 deaths worldwide per year due to intentional ingestions (Gunnell and Eddleston, 2003; Gunnell et al., 2007a). However, to the best of our knowledge, a severe case of PM poisoning with corresponding diagnostic investigations has not been published yet. Nowadays, due to stricter regulations, the death toll has decreased to approximately 100,000 deaths per year worldwide (Gunnell et al., 2007b; Mew et al., 2017). In 2015, a patient with severe PM intoxication was treated in a specialized toxicology unit at a tertiary care center of a university hospital in Germany. The case is described in the following.

**FIGURE 1:**
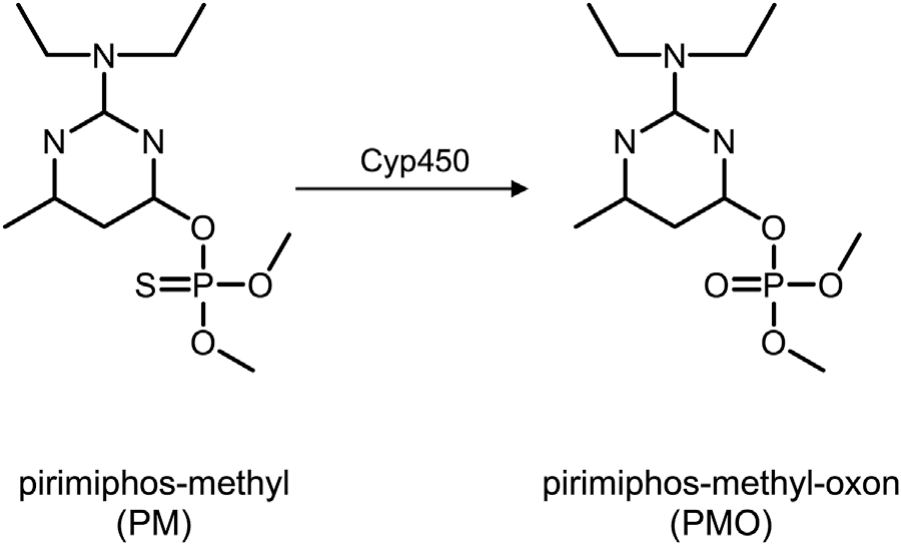
Pirimiphos-methyl (left) and pirimiphos-methyl-oxon (right) after metabolization *via* Cytochrome P450. Abbreviations: PM, pirimiphos-methyl; PMO, pirimiphos-methyl-oxon; Cyp450, Cytochrome P450.

As is typical for other OPs, the toxicity of PM is due to the phosphorylation of the enzyme acetylcholinesterase (AChE) which is inhibited by the OP (John et al., 2020). Phosphorylation in organophosphates describes the covalent binding of the phosphoryl moiety of the OP to any reactive center, e.g., at the side chains of certain amino acids, thus forming adducts (John et al., 2020). Similar to AChE, the circulating enzyme butyrylcholinesterase (BChE)—also known as pseudo-cholinesterase–is also a target of phosphorylation and inhibition (John et al., 2020). Prior to phosphorylation, the thio-pesticide PM (containing a P=S double bond) is transformed into its oxon-variant (containing a P=O double bond). *In vivo*, PM undergoes cytochrome P450-mediated desulfuration *via* cytochrome P450, yielding pirimiphos-methyl-oxon (PMO, Figure 1). This poison belongs to the class of oxono-pesticides which phosphorylate the serine residues of the active site of AChE (John et al., 2020). As a result, acetylcholine accumulates in the synaptic cleft of sympathetic and parasympathetic preganglionic synapses, central synapses, parasympathetic postganglionic synapses, and skeletal muscle afferent nerve endings. Accordingly, it stimulates muscarinic and nicotinic receptors causing a cholinergic crisis. For therapeutic intervention, phosphorylated AChE can be reactivated by oxime antidotes (e.g., obidoxime or pralidoxime) that cleave the phosphoryl moiety and restore the functional enzyme (John et al., 2020). Reactivation will be successful as long as the bound phosphoryl moiety has not already been “aged.” Aging is a chemical process of hydrolysis of at least one alkoxy group of the serine-bound phosphoryl moiety. Such aged adducts cannot be reactivated by oximes anymore, leaving the enzymes irreversibly inhibited (John et al., 2020). For biomedical analysis, adducts of BChE are analyzed, documenting exposure to OP (John et al., 2010a; John et al., 2020; John and Thiermann, 2021).

In addition, adduction of other serum proteins, especially of human serum albumin (HSA), is observed after OP poisoning (von der Wellen et al., 2018). To document poison incorporation, these adducts are proteolyzed with diverse enzymes yielding biomarker adducts, e.g., phosphorylated tyrosine (Tyr) (John et al., 2020; von der Wellen et al., 2018). Adducts of PM should yield a biomarker consisting of Tyr adducted with a dimethyl thiophosphate moiety (Tyr-DMTP), whereas PMO should yield Tyr adducted with a dimethyl phosphate moiety (Tyr-DMP). Such Tyr adducts can be monitored by liquid chromatography-electrospray ionization tandem-mass spectrometry (LC-ESI MS/MS), allowing their selective and sensitive detection.

## Materials and methods

### Patient data

Patient data were extracted from the hospital charts of the district hospital in Dachau, Germany, the emergency medical service (EMS) and the toxicological intensive care unit (ICU) of the Klinikum rechts der Isar, Technical University of Munich, Germany. Patient blood samples were analyzed at the respective clinical laboratories, and further samples were transferred to the Bundeswehr Institute of Pharmacology and Toxicology for specific analysis of the pesticides, cholinesterase status, and antidote concentrations in plasma. Patient consent for publication was obtained.

Results from the respective clinical laboratories were available to the treating physicians with the daily lab results. Data from the Bundeswehr were made available to the treating physicians as soon as measurements were completed.

Unfortunately, it was not possible to determine the exact time when the patient ingested the PM. Therefore, we chose either the admission to the tertiary care center or the time of the first blood sample as references for time, since these were the times which could be clearly determined.

### Cholinesterase and standard laboratory tests

BChE and standard laboratory tests (e.g., sodium, creatinine, leucocytes, C-reactive protein, pH, and lactate) were performed in the respective clinical laboratories.

### Sample storage and transfer

Patient EDTA blood samples were taken repeatedly, 0.2 ml was bedside diluted with 3.8 ml ice-cold distilled water, and plasma was collected after centrifugation. Whole blood dilution and plasma were immediately frozen (−20°C) until analysis. Blood was taken on admission and three times per day on the following days at 8 a.m., 4 p.m., and midnight.

### Detection of pesticides

PM and PMO were monitored by LC-ESI MS/MS in a similar way as described before, for analysis of the pesticides dimethoate and omethoate (John et al., 2010a).

### Detection of phosphorylated tyrosine adducts

Phosphorylated tyrosine (Tyr) adducts derived from modified plasma proteins predominantly from HSA were analyzed by micro liquid chromatography-electrospray ionization high-resolution tandem-mass spectrometry (µLC-ESI MS/HR MS) as described before (von der Wellen et al., 2018).

### Detection of BChE adducts

For analysis of phosphorylated BChE-derived nonapeptides, a µLC-ESI MS/MS method was applied as described before (John et al., 2015).

### Determination of cholinesterase status

AChE and BChE activity were determined with an UV-VIS spectrophotometer *via* modified Ellman assay as described before (Worek et al., 1999a), and the AChE and BChE activity, reactivatability of AChE and inhibitory activity of plasma were also measured (Worek et al., 2005; Worek et al., 2010).

### Determination of antidotes obidoxime and atropine

Obidoxime was measured *via* reversed-phase ion-pair chromatography-diode array detection (RPIPC-DAD) as described recently (Kranawetvogl et al., 2020). Atropine was monitored by LC-ESI MS/MS in multiple reaction monitoring mode (John et al., 2010b).

## Clinical case

A 63-year-old male was admitted to a district hospital after ingesting 700 ml vodka and two swallows of PM solution (500 g PM/L, Actellic® 50, Syngenta; approximately 25 g of PM) with suicidal intention.

His medical history included Child–Pugh Class B alcoholic liver cirrhosis with active alcoholism, esophageal varices grade II, status post epileptic seizure during alcohol withdrawal, adiposity, diabetes type II with cerebral microangiopathy and chronic renal insufficiency, necrotizing fasciitis of the left leg, and heparin-induced thrombocytopenia.

Upon arrival of the EMS, he was fully alert with no evident signs of cholinergic excess. Preclinically, EMS administered 50 g of oral activated charcoal and 0.5 mg atropine intravenously. He had one episode of vomiting. Arrival of the EMS on scene (6 p.m.) was approximately 3–4 h after ingestion (approximately 2–3 p.m.), and they brought the patient to the intensive care unit (ICU) of the district hospital approximately 4–5 h post ingestion of PM (transport time approximately 1 h).

Upon arrival at the district hospital as primary care center at 7 p.m., he received fluid resuscitation, another 25 g of activated charcoal, and further atropine (0.5 mg every 30 min) and was admitted to the local ICU. His blood ethanol level was 2 g/L. Arterial blood gas analysis showed an acidosis with a pH of 7.19 and highly elevated lactate of 25 mmol/L. He received sodium bicarbonate which resolved the acidosis, and the lactate was cleared with fluid resuscitation. On the next day, he was transferred to the ICU of our toxicology unit as tertiary care center and arrived there at 2:30 p.m.

Upon arrival at our ICU, the patient had no cholinergic signs (he presented with dry mucous membranes, warm skin, and mydriatic pupils), requiring only small doses of atropine. His initial vital parameters were stable (heart rate, 105/min; blood pressure, 155/75 mmHg; SpO_2_, 99% with 4L/min O_2_ *via* nasal cannula). Four hours after admission, he developed bradycardia and respiratory distress and was intubated. He received atropine by continuous infusion at a maximum dose of 10 mg/h for 7 days and obidoxime 250 mg as initial bolus and 750 mg/24 h as a continuous infusion for 7 days. Initially, epinephrine was used to stabilize the patient with a maximum dose of 500 μg/h; due to bradycardia, he repeatedly received dobutamine as continuous infusion within the following days (Table 1).

**TABLE 1:** Clinical course of patient from admission to primary care center until extubation, showing the clinical course of the intoxication. Blood pressure and heart rate are given as the lowest daily value, medications as their highest infusion rate on the respective day, O_2_ is displayed in liters per minute, and FiO_2_ in percent (also highest daily flow rates/percentages). Laboratory results from the respective clinical laboratories. Cumulative atropine = 248 mg (12 mg as bolus, 236 mg as continuous infusion), BP = blood pressure, HR = heart rate, CRP = C-reactive protein, CK = creatinine kinase, BChE = butyrylcholinesterase, NC = nasal cannula, BiPAP = bilevel positive airway pressure ventilation, FiO2 = fraction of inspired oxygen, and G = giga. Reference levels: sodium = 135–145 mmol/L; creatinine = 0.7–1. mg/dl; CK<174U/L; leucocytes = 4.0-9-0G/L; CRP<0.5 mg/dl; BChE = 5320–12920U/L; pH = 7.35–7.45; lactate<2.4 mmol/L.

Day	-1	0	1	2	3	4	5	6	7	8	9	16
Events	Admission to district hospital	Admission to tertiary care center, cholinergic crisis, intubation				Cholinergic crisis						Extubation
BP (mmHg)		95/50	100/55	120/55	110/50	125/55	130/70	125/55	130/55	100/55	110/55	90/40
HR (/min)		52	60	60	60	55	55	50	60	60	55	70
Respiration (liters per min or FiO_2_)		4 L/min NC/BiPAP 40%	BiPAP	BiPAP	BiPAP	BiPAP	BiPAP	BiPAP	BiPAP	BiPAP	BiPAP	BiPAP 40%/4 L/min NC
			40%	40%	40%	40%	40%	40%	40%	40%	40%	
Sodium (mmol/L)	121	143	142	139	144	143	146	148	150	149	149	146
Creatinine (mg/dl)	1.1	1.1	1.4	1.6	1.3	1.1	1.0	1.0	1.1	1.1	1.2	1.4
CK (U/L)	171	222	145	2090	6398	3215	1231	500	291	209	267	50
Leucocytes (G/L)	14.7	9.3	14.2	7.2	5.5	5.0	5.5	5.2	4.5	5.3	5.9	6.5
CRP (mg/dl)	0.3	-	1.0	3.5	6.7	6.6	5.2	4.5	4.1	4.1	4.7	1.7
BChE (U/L)	480	118	105	141	194	261	512	734	924	1057	1248	2085
pH	7.19	7.52	7.33	7.48	7.46	7.41	7.41	7.40	7.40	7.40	7.47	7.40
Lactate (mmol/L)	25	2.1	-	1.4	0.9	-	-	-	1.2	-	-	-
Atropine (mg/h)	1.0	9.5	4.5	6.5	1.5	4.5	1.5	3.5	1	0.2	-	-
Atropine bolus (mg)	0.5	0.5		5.0		3.0		3.0				
Obidoxime (mg/h)	-	31.25 mg/h plus 250 mg bolus	30	30	30	30	30	30	30	30	-	-
Epinephrine (µg/h)	-	500	500	200	-	-	-	-	-	-	-	-
Norepinephrine (µg/h)	-	300		-	-	-	-	-	-	-	-	-
Dobutamine (mg/h)	-	-	-	15	18	9.3	9	9	8.5	5	-	-

The subsequent clinical course was complicated by bouts of pneumonia, necessitating prolonged ventilatory support. Extubation was possible on day 16 after admission to our ICU; however, re-intubation and tracheotomy were necessary. The further course was complicated by severe delirium and catheter-associated sepsis with a septic shock, which required mechanical ventilation for another 17 days (week 4 and 5).

Due to profound peripheral neuropathy, the patient was discharged—after psychiatric evaluation—to a neurological rehabilitation facility after 77 days of cumulated ICU and hospital care.

## Results

### Detection of pesticides

PM was detectable in plasma immediately and showed its maximum concentration ∼18 h post admission to our ICU. It was still detectable 160 h post admission.

**FIGURE 2:**
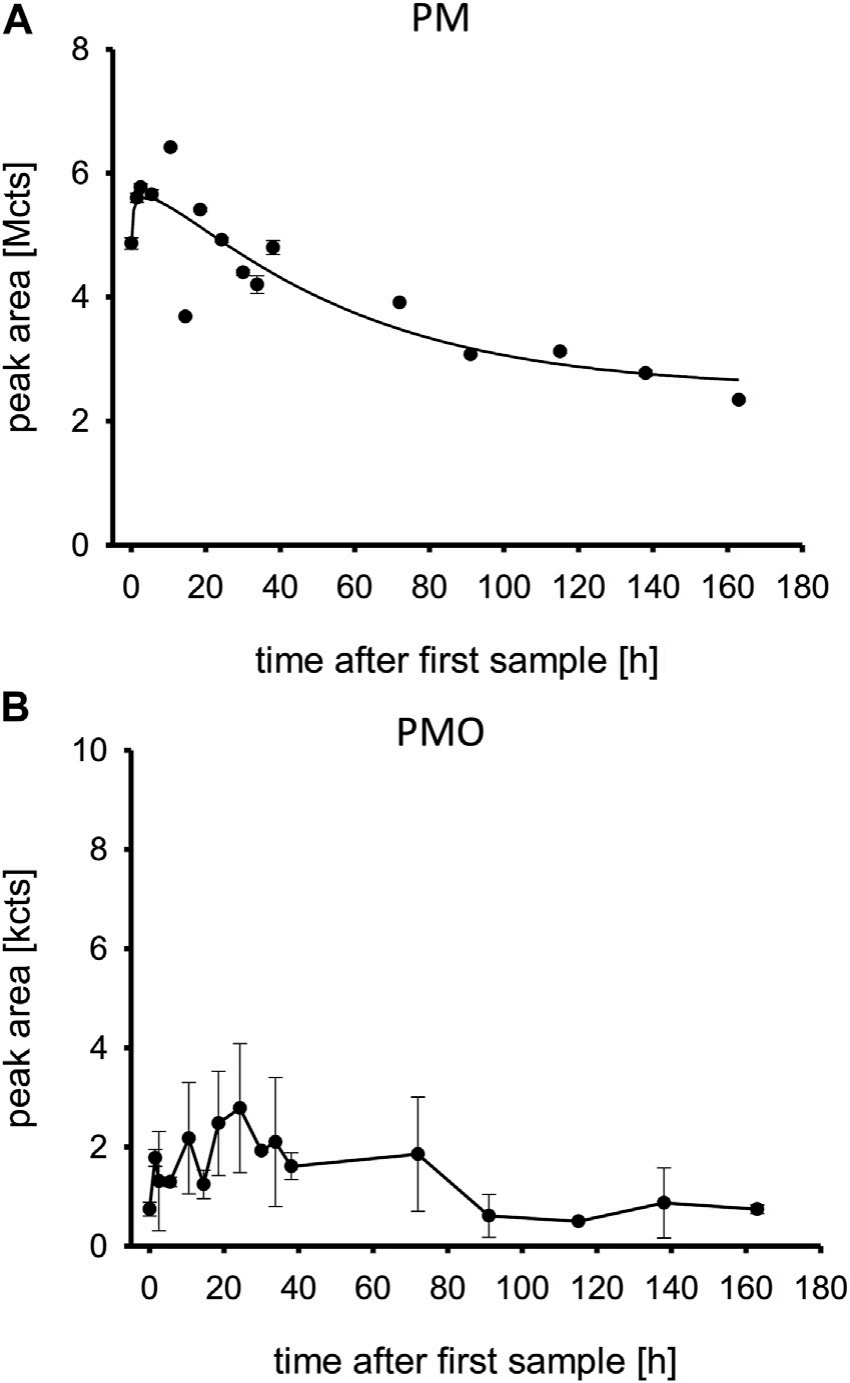
**(A,B)** Pirimiphos-methyl and pirimiphos-methyl-oxon in patient plasma. Time is shown in hours after first blood sample, which was approximately upon admission to the tertiary care center ICU. Abbreviations: PM, pirimiphos-methyl; PMO, pirimiphos-methyl-oxon; Mcts, mega-counts; kcts, kilo-counts.

The highest peak area was at ∼10 h after first sample, half of the highest peak area was reached at ∼90 h after first sample. Therefore, the approximated half-life in this overdose was calculated at 80 h (Figure 2A). PMO was also detectable on admission and peaked at ∼18 h post admission. It also was still detectable at 160 h post admission (Figure 2B).

### Detection of Tyr adducts

**FIGURE 3:**
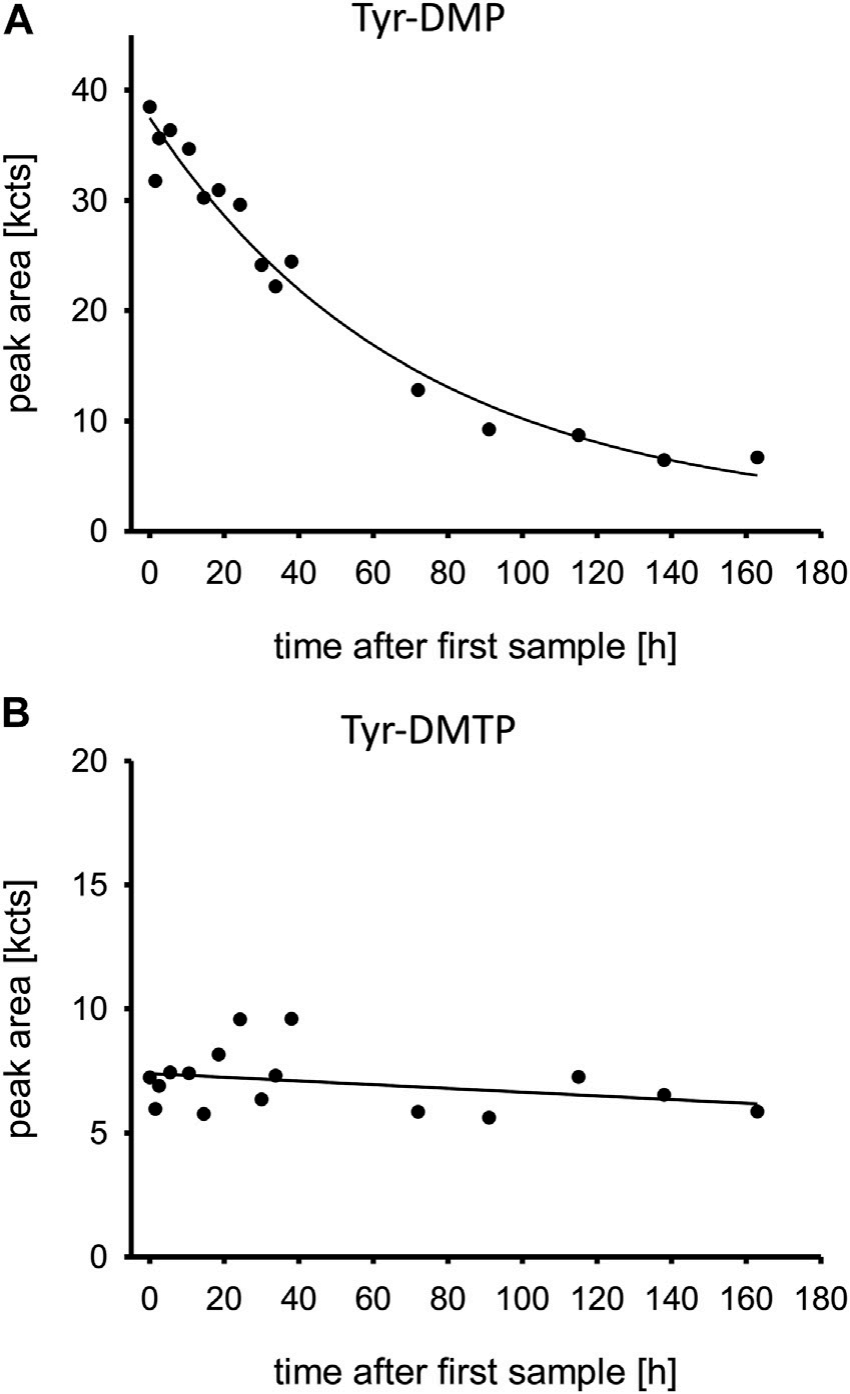
**(A,B)** Oxono-tyrosine and thiono-tyrosine adducts to human serum albumin. Time is shown in hours after first blood sample, which was approximately upon admission to the tertiary care center ICU. Abbreviations: Tyr-DMP, tyrosine dimethyl thiophosphate moiety; Tyr-DMTP, tyrosine dimethyl phosphate moiety; kcts, kilo-counts.

The relative concentration-time profiles of the Tyr-DMP and Tyr-DMTP are displayed in Figure 3. While Tyr-DMP peaked early and decreased over time, Tyr-DMTP remained at a steady state in very low relative concentrations during the first 160 h post admission.

### Cholinesterase

BChE was already suppressed at admission in the primary care center; the BChE measured by the clinical laboratory is shown in Table 1. After having passed its nadir on day 1 after admission to the tertiary care center, termination of the antidote treatment was possible after BChE had recovered to 1/5th of its physiological value. Extubation was possible after BChE had recovered to 2/5th of its normal value (Table 1).

### Determination of cholinesterase status

**FIGURE 4:**
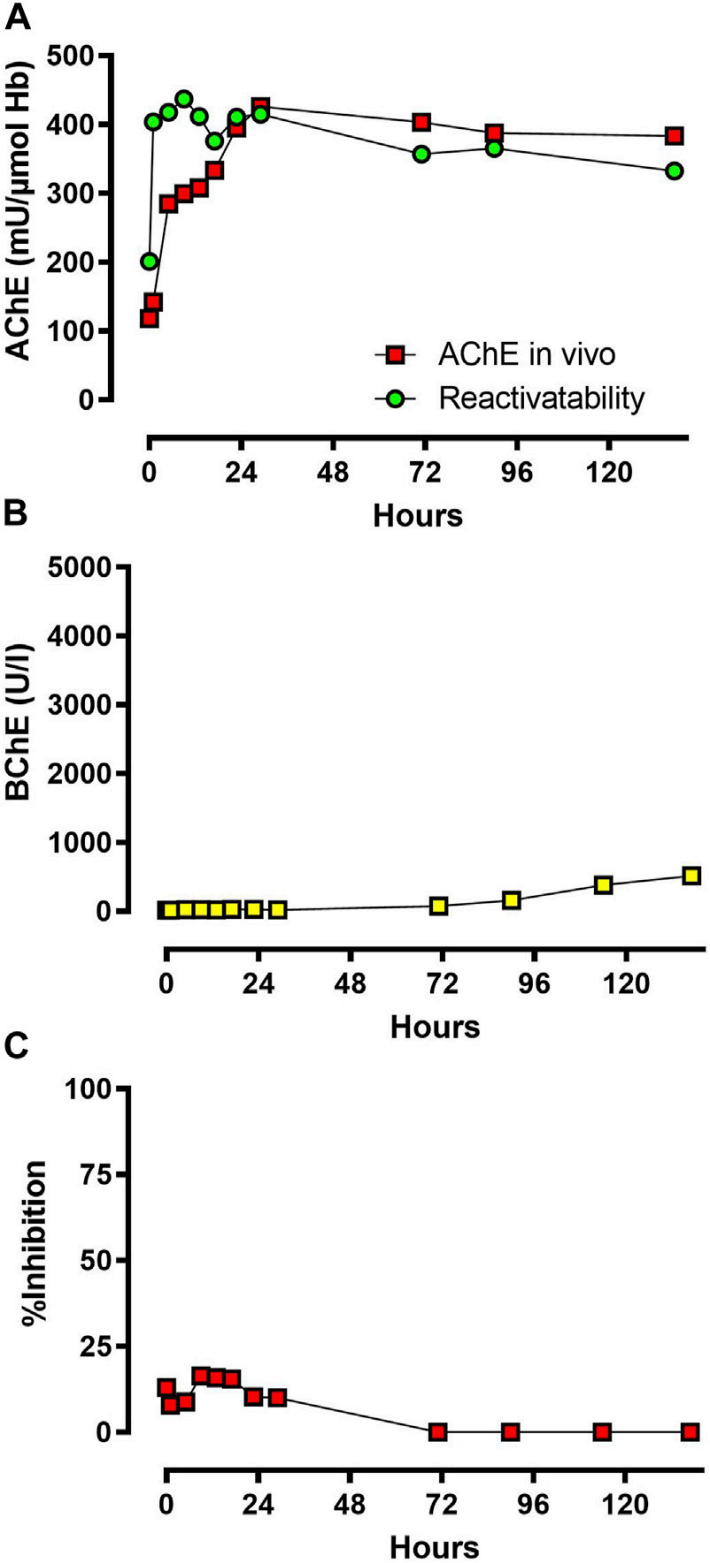
**(A,B)** and **(C)**: AChE *in vivo* and reactivatability, BChE *in vivo*, and inhibitory activity of patient plasma. Time is shown in hours after admission to the toxicological unit. Abbreviations: AChE, acetylcholinesterase; BChE, butyrylcholinesterase.

AChE *in vivo* and its reactivatability are shown in Figure 4A, including the inhibitory activity of the patient’s plasma (Figure 4C). AChE *in vivo* was suppressed at admission, increased continuously after start of obidoxime therapy, and reached its maximum activity after ∼30 h. AChE *in vivo* and reactivatability remained at the same level until the end of monitoring of the enzyme activities.

BChE activity measured by UV-VIS spectrophotometer *via* modified Ellman assay was markedly suppressed at admission and increased slowly after ∼70 h, which correlates with the gradual decrease of inhibitory activity (Figure 4B).

### Detection of BChE adduct

**FIGURE 5:**
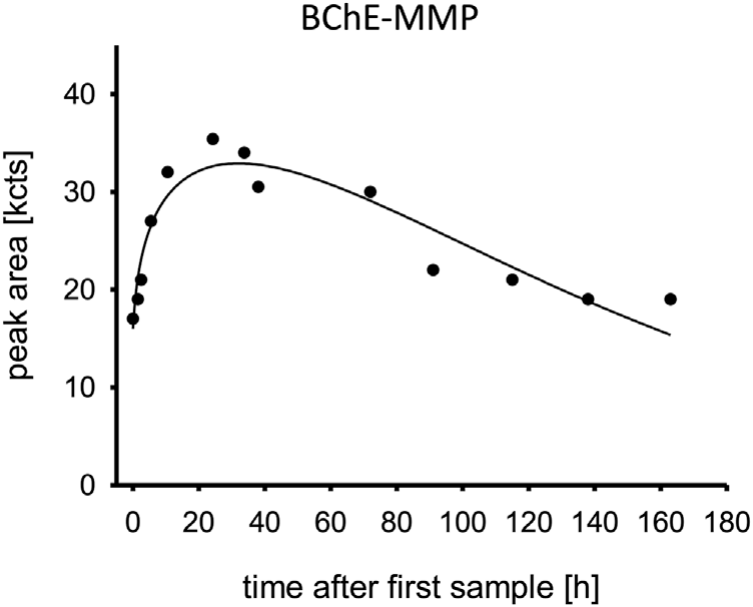
Aged butylcholinesterase. Time is shown in hours after first blood sample, which was approximately upon admission to the tertiary care center ICU. Abbreviation: BChE-MMP, butylcholinesterase monomethyl phosphate moiety; kcts, kilo-counts.

The serum levels of the aged BChE adduct containing a bound monomethyl phosphate moiety (BChE-MMP) are shown in Figure 5. While a substantial part of the BChE was already aged at admission, the aging continued with a peak at 24 h post admission and slowly declined afterward. Surprisingly, the non-aged adduct was not detected.

### Determination of antidotes obidoxime and atropine

**FIGURE 6:**
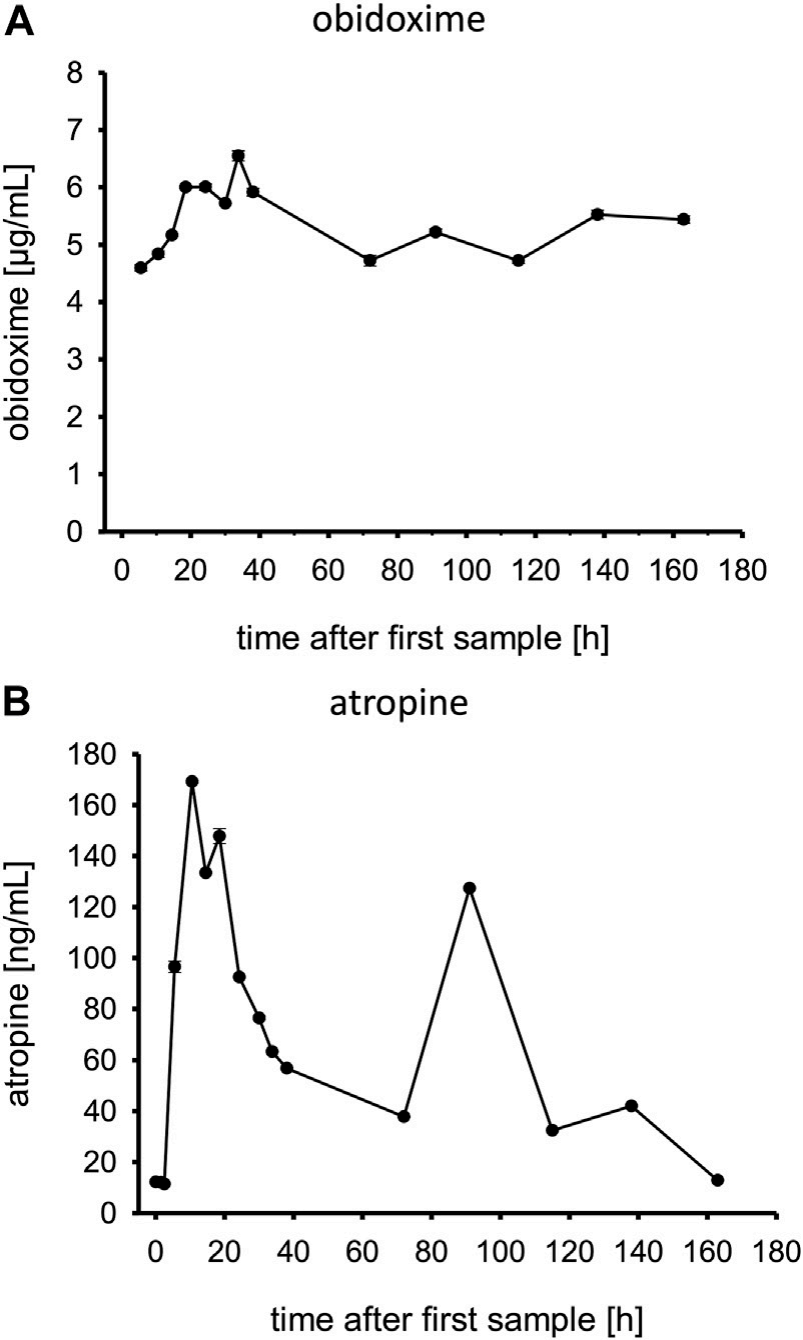
**(A,B)** Obidoxime and atropine levels in patient plasma. Time is shown in hours after first blood sample, which was approximately upon admission to the tertiary care center ICU.

Obidoxime levels are displayed in Figure 6A. After the obidoxime bolus of 250 mg, it further increased under continuous infusion until it reached a steady state. This continued until the obidoxime infusion was stopped. On admission, plasma atropine levels were low, corresponding with the applied low doses of 0.5 mg every 30 min as shown in Figure 6B. The plasma level increased with the start of the continuous infusions of 9.5 mg/h. Afterward, the level dropped to a steady state until atropine infusions were discontinued.

## Discussion

### Initial presentation and onset of symptoms

Upon admission to the primary care center and upon transfer to the tertiary care center, the patient did not shown a full cholinergic syndrome, though treatment with atropine was necessary with initially rather low doses compared to the usually applied doses for severe organophosphate poisoning. Treatment with obidoxime was not initiated by the district hospital due to the lack of cholinergic signs and unavailability of this antidote. According to the clinical signs, the development of severe cholinergic crisis took more than 27 h after ingestion of PM, indicating a slow decrease of AChE activity. On admission, AChE activity was markedly but not completely inhibited, which fits the clinical picture. Obidoxime was chosen as an antidote in combination with atropine since it has been shown to be safe and a potent reactivator of OP-inhibited AChE in other cases of OP poisoning (Eyer et al., 2009).

### Antidote therapy: Obidoxime and atropine

An obidoxime bolus and continuous infusion over 7 days resulted in a rapid increase of AChE activity which remained at a sufficiently high level. We applied obidoxime in the standard dosing regimen. The plasma obidoxime levels in steady state reached ∼5 μg/ml (approx. 15 μmol/L) which is the recommended level (Figure 6A) (Eyer et al., 2009).

Atropine plasma levels correlated well with the given atropine doses. At ∼90 h post admission to the tertiary care center, the patient developed another episode of cholinergic syndrome, requiring increased atropine infusion rates and an atropine bolus (Table 1) which can also be seen in the plasma levels (Figure 6B). Ultimately, it remains unclear why the patient had renewed cholinergic symptoms after about 90 h, which resulted in a temporary increase in dosage of atropine. In any case, neither the cholinesterase status (AChE; BChE) nor the concentrations of PM or PMO at that time suggest that there was a significant increase in the load of OP (e.g., late reabsorption) that justified a renewed more intensive therapy with atropine. The decision to adjust the atropine dosage was made clinically, without awareness of the analytical results from, e.g., PM or PMO.

In general, the patient required prolonged atropine application with high continuous infusion rates and repeated boluses in high doses. The cumulative applied atropine was 248 mg (12 mg as bolus, 236 mg as continuous infusion), which is unusually high for an OP intoxication and could be a noteworthy peculiarity in PM poisoning. In OP poisoning, atropine is dosed according to clinical symptoms. Those high doses usually show no relevant side effects, since all atropine is required to antagonize the muscarinergic effects of the OP. Our patient never experienced episodes of over-atropinization, such as tachycardia or other anticholinergic symptoms, despite those high doses.

AChE activity was analyzed off-site, and the results were reported to the clinic with some delay. Therefore, the discontinuation of obidoxime was decided by the clinical signs. Determination of BChE activity is generally used in hospitals since assays for AChE activity are usually not available. In these cases, an increase of BChE activity indicates the absence of an inhibitor in blood which is one parameter for deciding to stop oxime therapy.

As expected, obidoxime had no effect on BChE activity, which increased only after inhibitory activity was absent due to *de novo* synthesis. Spontaneous reactivation of dimethyl-OP-inhibited BChE is possible at a rather low rate. However, in this case, BChE activity increased (by *de novo* synthesis and spontaneous reactivation) only after loss of inhibitory activity, i.e., presence of PMO, starting on day 3 (Worek et al., 1999b). The Tyr-DMP concentration decreased in the days post admission, the patient plasma still had inhibitory activity (remaining pesticide), and aging of the BChE continued despite treatment with obidoxime. Even though BChE showed significant aging, reactivatability of AChE was nevertheless rather high, suggesting that BChE has a limited clinical relevance.

### Pesticides

The high concentration of PM in comparison to PMO documents its high resistance in the circulation and a notably low biotransformation process to PMO, potentially caused by impaired liver activity due to the patient’s underlying Child–Pugh Class B alcoholic liver cirrhosis. The low relative concentrations of PMO compared to PM document that most of this PM biotransformation product has been either eliminated by urine or reacted with diverse endogenous molecules including, e.g., cholinesterases and HSA.

Our measurements documented that although the patient did not yet show severe signs of cholinergic crisis, he already had high plasma pesticide levels (Figure 2A), and a large amount of the BChE had already aged irreversibly (Figure 5). The AChE was initially suppressed, but it still showed reactivatability and rapidly increased after treatment with obidoxime was started. AChE reached its maximum 30 h post treatment initiation. Consequently, the patient’s clinical condition initially deteriorated, and he required mechanical ventilation and circulatory support with catecholamines. Our laboratory measurements cannot sufficiently explain the renewed cholinergic crisis at day 4.

### Tyr and BChE adducts

The quite rapid decrease of Tyr-DMP was unexpected as phosphorylated Tyr from HSA was thought to be stable for some weeks as determined by the natural protein turn-over (John et al., 2020). Accordingly, the decrease of this DMP adduct might have been due to spontaneous hydrolysis (cleavage of the entire phosphoryl moiety) or any process of enzymatic dephosphorylation not unraveled so far. The low concentrations of Tyr-DMTP illustrate a low reactivity with HSA despite the high PM concentrations. In addition, the rapid decrease of Tyr-DMP may also be caused by pathological renal loss of HSA in general.

The rapid decrease of the BChE-MMP concentration is explained by the natural protein turn-over (John et al., 2020) and the spontaneous reactivation yielding functional BChE (Worek et al., 2005; Worek et al., 2010). The kinetics of aging of AChE and BChE are different, leading to different levels of aged AChE and BChE (Worek et al., 1999b).

### Clinical course

Even though the laboratory findings improved with treatment, the patient’s condition remained critical for 4 days, requiring high doses of atropine and only improved slowly afterward. He had a complicated further clinical course, also partly due to his comorbidities. His initial pneumonia was due to aspiration and resolved slowly despite broad spectrum antibiotic treatment and lung protective ventilation and required tracheotomy. Nevertheless, he may have benefitted from an earlier application of obidoxime, although there is still a controversial debate about the clinical impact of oxime therapy alongside best supportive critical care, including atropine (Eddleston, 2022).

Although the acute toxicity of organophosphates is largely explainable by AChE inhibition and the lasting effects of poisoning by direct excitotoxicity or indirect consequences of the cholinergic syndrome, effects at lower levels of exposure could not be predicted from this mechanism. Carter et al. found significant adduction of partially characterized protein targets in both rat brain and thymus by azamethiphos, chlorfenvinphos, chlorpyrifos-oxon, diazinon-oxon, dichlorvos, and malaoxon, *in vitro* and PM *in vivo* (Carter et al., 2007).

## Limitations

As with all case reports, this case shows data from only one patient, so it is difficult to draw general conclusions from it.

Laboratory findings by the Bundeswehr Institute of Pharmacology and Toxicology were made available to the treating physicians with some delay (inherent to the complex analytical process); this could have influenced their clinical decision-making if they had been available earlier.

## Conclusion

We describe a case of severe PM intoxication with a complicated and prolonged clinical course. Our patient required rather high doses of atropine. Despite delayed obidoxime treatment, AChE activity recovered to large extent. The support by a specialized laboratory allowed the determination of the cholinesterase status (AChE and BChE activity, reactivatability, and inhibitory activity of the patient’s plasma) and the analysis of BChE and albumin adducts to verify the intake of PM and its conversion into PMO. While these measurements were not available in time for treatment decisions, which were made clinically, they give a comprehensive insight into a rather rare poisoning. For optimal care, patients with organophosphate poisoning should be admitted to a hospital specializing in clinical toxicology.

Our clinical and analytical finding in this rare case of PM poisoning suggests the following:

- Patients with PM poisoning should be observed in an ICU for at least 48 h because a delayed onset of cholinergic crisis is possible with this agent
- PM has an *in vivo* half-life of ∼80 h in overdose
- AChE could have been reactivated by antidote therapy in this case, even though the antidote was not given earlier than 24 h post ingestion

## Data Availability

The datasets for this article are not publicly available due to concerns regarding participant/patient anonymity. Requests to access the datasets should be directed to the corresponding author.

## Abbreviations

AChE, acetylcholinesterase; BChE, butyrylcholinesterase; BChE-MMP, aged BChE adduct containing a bound monomethyl phosphate moiety; EMS, emergency medical service; h, hours; HSA, human serum albumin; ICU, intensive care unit; LC-ESI MS/MS, liquid chromatography-electrospray ionization tandem-mass spectrometry; µLC-ESI MS/HR MS, micro liquid chromatography-electrospray ionization high-resolution tandem-mass spectrometry; mg, milligram, milligrams; OP, organophosphate; p.a., post admission; PM, pirimiphos-methyl; PMO, pirimiphos-methyl-oxon; RPIPC-DAD, reversed-phase ion-pair chromatography-diode array detection; Tyr, tyrosine; Tyr-DMP, tyrosine adducted with a dimethyl phosphate moiety; Tyr-DMTP, tyrosine adducted with a dimethyl thiophosphate moiety.
